# Redefining Representativeness of a Sample in Causal Terms

**DOI:** 10.1111/jep.70137

**Published:** 2025-06-23

**Authors:** Michał Sikorski, Alexander Gebharter, Barbara Osimani

**Affiliations:** ^1^ Center for Philosophy, Science, and Policy Marche Polytechnic University Ancona Italy

## Abstract

**Rationale:**

Despite its crucial role, sample representativeness remains a controversial topic in the methodology of medical science. There is an ongoing debate not only about how best to define and ensure the representativeness of a sample (e.g., Rudolph et al. 2023; Porta 2016), but also about whether representativeness is worth pursuing at all (e.g., Rothman et al. 2013).

**Aims and Objectives:**

Our aim is to construct a formalised, precise, and practical conceptualisation of sample representativeness.

**Methods:**

We employ the established framework of causal Bayesian networks to develop such a conceptualisation.

**Results:**

We propose a precise formal definition of sample representativeness that translates into clear and actionable methodological guidance. Additionally, we provide examples and a checklist to illustrate the application of the proposed conceptualisation.

**Conclusion:**

We believe that the presented definition will facilitate further discussion of the issue of representativeness and prove useful to scientists in practice.

## 1 Introduction

Because in typical medical experiments it is not feasible to test all members of the target population, scientists rely on samples. This requires that the sample represents the target population at least to some degree. Surprisingly, despite its crucial role, representativeness remains a controversial topic within the methodology of medical science. It remains unclear not only how to best define and ensure representativeness, but also whether it is worth pursuing in the first place. In this paper we present a new definition of representativeness in terms of causally interpreted Bayesian networks (CBNs). We argue that this new definition is not only more precise, but also provides more guidance than existing alternatives.

We critically discuss the accepted definitions of representativeness in Section 2. In Section 3, [Link], we introduce CBNs and our new definition of representativeness. In Section 4, we compare our proposal to already existing alternatives.

## 2 Existing Definitions and Their Deficiencies

In a recent article Rudolph et al. [1] discuss two definitions of representativeness used in medical science and propose a new one. In this section, we describe all three proposals and highlight some of their shortcomings. We assess the proposals with respect to how adequate they are—that is, how well they capture the concept of representativeness in the way it is typically used—and their potential for guiding scientists in their search for representative samples.

### 2.1 Representativeness as Random Sampling

According to the first definition, a sample is representative if

**RR** ‘[…] the study sample is a simple random sample of the target population (i.e., the sample that arises through representative sampling)’

(Rudolph et al. [1] p. 1)

Randomisation secures the representativeness of a sample with a high probability if each unit of the sampled population has the same probability of being selected. Formally, if it can be assumed that ‘sample draws’ are independent and identically distributed or, at least, exchangeable (see [2] or [3]). Despite that, the desirability of representativeness along the lines of **RR** is controversial. Rothman et al. [4] argued that representative samples should be avoided. It is often more fruitful to study many nonrepresentative samples (e.g., w.r.t. age categories) because this might show an effect that could be hidden when data are pooled into a single representative sample. The majority of the commentators agreed with the conclusions of Rothman et al. They argued that simple random sampling is often costly to the point of being impossible and that the results achieved in nonrepresentative samples are often generalisable (e.g., [5, 6, 7]). Surprisingly, none of these authors argued for revising the definition of representativeness rather than abandoning the requirement of representativeness. A lesson one may draw from this discussion is that randomisation is not necessary for representativeness. Also note that **RR** is only applicable to cases in which the sample is a subset of the target population. This implies that **RR** does not apply to extrapolation where the study sample is by definition not drawn from the target population [4]. Samples composed of nonhuman model organisms, fragments of tissues, or computer simulations, for example are paradigmatic in this respect, since they cannot be immediately applied to a target population composed of humans. But also without crossing boundaires in levels of inference, results from studies performed on a given population do not necessarily apply to other populations (for socio‐anagraphic factors or other relevant sets of conditions). This seems to be inconsistent with how extrapolation of results across species is understood in science. Thus, **RR** may at best be a good definition of a specific kind of representativeness. Finally, what lessons for obtaining a representative sample can be learned from **RR**? To obtain a representative sample, one needs to endorse randomisation procedures which, as the extensive discussion in [4] and commentators shows, is often expensive, time‐consuming, and in some cases impossible. Thus, collecting a random sample might be the right approach in some cases, but seems to be unpractical as a general strategy.

### 2.2 Representativeness as Similarity

Another definition discussed by Rudolph et al. [1] characterises representativeness in terms of similarity:

**SR1** ‘[…] the study sample and the results obtained merely resemble what would be expected in the target population, perhaps based on a similarity in personal characteristics’.

(Rudolph et al. [1] p. 1)

The source of this definition is a dictionary of epidemiology [8]. Thus, it seems likely that this or a similar definition is widely assumed by researchers. The main problem with this definition is that it is underspecified. It is not clear which personal characteristics should be considered. If all of the possessed properties should be considered, then **SR1** seems to collapse to **RR**, as using a randomised sample seems to be the best way to reliably reproduce the distribution of properties among individuals of the target population. Consequently, **SR1** would share all the deficiencies of **RR**. If, on the other side, only some of the characteristics should be considered, then which properties are the relevant ones?

What are the practical consequences of **SR1**? The definition seems to suggest that scientists should look for a sample that (a) shears some of the characteristics of the target population and that (b) delivers results similar to those that would be expected in the target population. This does not seem to be particularly helpful for at least two reasons. Firstly, as already mentioned, it is not clear what characteristics are relevant. Secondly, to satisfy (b) scientists need to determine if the results reached in the sample population will resemble the effects that would be obtained in the target population. Since this assessment is difficult before having carried out the study, also **SR1** does not seem to give useful advice.

The newest edition of the *Dictionary of Epidemiology* [9] presents a slightly different definition which is worth citing in full:

**SR2** ‘REPRESENTATIVE SAMPLE A sample that to a large extent resembles a population of interest. The term *representative* as it is commonly used is largely undefined in the statistical or mathematical sense. The use of probability sampling will not ensure that a sample will be representative of the population in all relevant aspects. It is unwarranted to assume that if the sample resembles the reference population on factors that have been checked, no differences exist in other relevant factors’

(Porta, [9] p. 247)

The comparison between **SR2** and **SR1** as paraphrased in [1] reveals interesting differences. Firstly, the results are not mentioned in the definition and therefore subclause (b) cannot be derived from the new version. Thus, the definition avoids the second problem of the earlier version. The definition is, though, still underspecified. Hence, also **SR2** does not provide significant guidance to scientists. Additionally, **SR2** mentions that the concept of representativeness is not well defined and that using probability sampling does not warrant representativeness. This does not seem to apply to the random simple samples described above but to other probabilistic sampling methods such as cluster sampling, or stratified sampling that employs some of the properties to group participants.

### 2.3 Rudolph et al.'s Definition

A new definition developed by Rudolph et al. [1] is an improved version of a definition in terms of similarity:

**GR** ‘We define a study sample to be representative of a well‐defined target population if the results estimated in that sample are generalisable to the target population’.

(Rudolph et al., [1] p. 1)

The definition captures the purpose behind collecting representative samples. Because it is usually not possible to check for each member of the target population whether the effect of interest occurs, we need such a sample as a basis for projecting the actually observed effect to the target population. Therefore, it seems to be almost trivially true that all samples should be representative in this sense. **GR** was partly motivated by the discussion of **RR** and succeeded in providing a much less controversial definition.

However, **GR** does not provide much guidance to scientists in their search for a representative sample. Though it highlights the necessity of an explicit description of the target population (further developed in the paper), it remains unclear how to determine whether the results observed in the sample can be generalised and to what degree they can be projected to the target population.

## 3 A Causal Analysis of Representativeness

**Figure 1:**
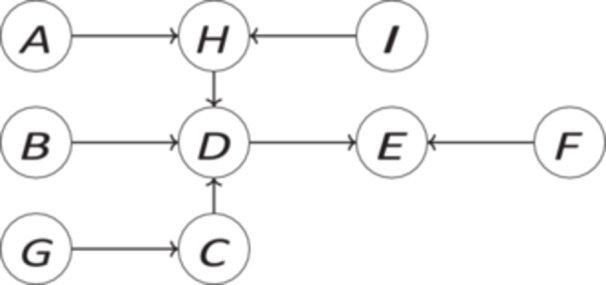
Simple exemplary causal model.

In this section, we reconstruct the debate concerning the desirability of representativeness (understood as in **RR**) on the basis of causally interpreted Bayesian networks (CBNs). Graphical causal models such as CBNs and causal structural equation models (SEMs) systematically link complex causal structures to probabilistic dependence. They can be used for representing causal structures, predicting causal effects based on observation and intervention, and inferring causal structure based on observational and experimental data^1^ (for details on CBNs see, e.g., [10, 11]). From a mathematical point of view, they do not only constrain the set of joint probability distributions that can be observed on the assumption that they reliably represent the data generating process, but also the probability distribution that each variable can take, based on interventions on its putative causes. Hence, they are crucial for disambiguating issues of representativeness, which, as a matter of fact, as the aforementioned debate testifies, does not only hinge on the distance between joint distributions of variables in samples vs. target populations, but also on their structural relations. From a causal perspective, both proponents and critics of **RR** are partially right. To see why, let us construct a simple CBN 〈**V**, **E**, *Pr*〉 featuring the variables *A, B, C, D, E, F, G, H, I*. We assume that the model's graph is the one in Figure 1 and that all direct causal relations (represented by arrows) indicate positive causes, meaning that the higher the values of the cause variables, the higher the probabilities for higher values of the effect variables. In addition, we assume that *A, B, C* are interactive effect modifiers w.r.t. *D*. In particular, we assume that they boost each others’ influences on *D*, meaning that the higher the values of each of these variables are, the stronger their individual and joint influence is on *D*.

**RR** implies that all features of the individuals in a representative sample are distributed among these individuals as they are distributed among the individuals of the target population. Let us assume that our study's goal is to investigate the effect of *A* on *D*. Let us further assume that we have a sample *s*_1_ in which the values of all nine variables are indeed distributed among the individuals as they are in the target population. Let us also assume that *B*‐ and *C*‐ values are skewed towards very low values in the target population. As a consequence, most units in the sample appear to manifest very low values for *B* and *C*. Thus, carrying out our study based on *s*_1_ will result in a weak positive effect of *A* on *D*. This effect can be expected to be the same as in the target population (random error excluded). Note that for this result it does not matter whether we measured any of the variables except *A* and *D* because due to randomisation it can be assumed that, excluding sampling errors, the distributions of covariates are probabilistically equivalent in the two populations.

Let us now consider an alternative sample *s*_2_. We assume that *s*_2_ differs from *s*_1_ insofar as *B*‐ and *C*‐values are much more equally distributed among individuals than they are in *s*_1_ and in the target population. As a consequence, we can expect that *G*‐ and *E*‐values are on average higher (since *G* and *D* are positive causes of *C* and *E* respectively) and that *D*‐values are on average higher (because we assumed that *A, B, C* boost each other's influence on *D*). *s*_2_ is clearly not representative of the target population according to **RR**. Now we can reconstruct Rothman et al.'s 2013 observation in terms of our simple model. In particular, we can specify a specific causal scenario in which a nonrepresentative sample seems clearly better than a representative one. Based on *s*_2_ we can learn more about *A*'s potential effect on *D* and about how to optimally utilise *A* for controlling *D* than we can learn from the representative sample *s*_1_. The reason is that, unless the size of the sample is large enough, the rareness of individuals with other than very low *B*‐ and *C*‐values in sample *s*_1_ does not allow us to condition on these other *B*‐ and *C*‐values because we do not have enough data to reliably infer how these *B*‐ and *C*‐values would influence *A*'s efficacy w.r.t. *D*. Not so in the nonrepresentative sample *s*_2_. Since in this sample we find all *B*‐ and *C*‐values instantiated sufficiently often by individuals, we have access to this information and are able to obtain a richer understanding of the counterfactual dependence of *D* on *A* given different values of *B* and *C*. This speaks in favour of Rothman et al. Note that uncovering the patterns of how *D* counterfactually depends on *A* requires that *B* and *C* have been measured.

Which sample is better, *s*_1_ or *s*_2_? We think that depends. Rothman et al.'s 2013 argument appears to conflate two distinct purposes a sample might have: (i) making a reliable inference about *A*'s effect on *D* in the target population versus (ii) understanding how *D* counterfactually depends on *A* when varying other factors such as *B* and *C*. Sample *s*_1_ allows for (i), but not for (ii) due to *B*‐ and *C*‐values, other than very low ones, being underrepresented in *s*_1_. Sample *s*_2_, on the other hand, allows for (ii) but, at least without further information about the target population, not for (i). While sample *s*_1_ being representative (according to **RR**) guarantees that any observed effect of *A* on *D* in the sample will be the same in the target population, *s*_2_ does not. Since in *s*_2_ *B*‐ and *C*‐values are distributed differently than in the target population (by assumption), it can—though allowing us to study how *A* would hypothetically influence *D* to the background of different *B*‐ and *C*‐values—not be used to make reliable predictions about *A*'s effect on *D* in the target population as long as we do not know in addition how exactly *B*‐ and *C*‐values are distributed in the target population. Thus, which type of sample to prefer depends on one's specific goals: inference (estimation of a causal effect) versus understanding (e.g., analysis of mediating/moderating factors). Hence, the analysis shows that certain samples such as *s*_1_ are unsuitable for generating understanding in terms of counterfactual dependence, but does not provide evidence that a nonrepresentative sample is generally preferable. Representative samples seem necessary to ensure generalisable results, at least as long as the distribution of other direct causes of the purported effect of interest is not known for the target population, in which case a nonrepresentative sample such as *s*_2_ is always preferable. Since this information is usually not available for the target population and this is the context in which representativeness actually does matter, we will assume for the remainder of the paper that such knowledge about the distribution of other factors in the target population is not available to the scientist.

Moreover, the example demonstrates that **RR** is unnecessarily strong. One does not have to have all variables' values distributed in the sample as they are distributed in the target population. For example, in a study investigating the effect of *A* on *D*, other direct causes of *D* not lying on a path from *A* to *D* such as *B* and *C* should be distributed in the sample as they are in the target population, as we just saw. Also other variables that might distort *A*'s impact on *D* due to influencing causes of *D* that are at the same time effects of *A* such as *I* need to be distributed equally. All other variables can in principle be distributed differently (see also recent graph theory contributions to the topic [12, 13, 14, 15]). More distant causes of direct causes of *D* such as *G*, for example, do not matter for *A*'s impact on *D* in the target population as long as possible disturbing factors such as *B*, *C*, and *I* are equally distributed in the sample and the target population. The same goes for *A*, *H*, and *D* because we control for *A* in the experiment and *H*'s probability is determined by *A* and *I* while *D*'s is determined by its direct causes *H*, *B*, and *C*. Thus, bringing about *A* in the population will have the same effect on *D* as it has in the sample as long as *B*, *C*, and *I* are equally distributed. The same reasoning as for *D* applies to effects such as *E*. Finally, variables that are not causes of *D* such as *F* can also be distributed differently since their distribution will not play any role in evaluating *A*'s impact on *D*.

Summarising, if one wants to infer a variable's causal impact on another one in the target population and does not have additional knowledge about how the effect's direct causes are distributed in the target population, a representative sample is necessary. But not a fully representative sample in the sense of **RR**. We only require causes of the effect variable that might distort *A*'s impact on *D* to be distributed equally in the sample as they are in the target population. Ultimately, representativeness is a relational property that depends both on the joint distributions of variables in the sample and target populations, but also on the scope of the (causal) association(s) being extrapolated from one to the other.

### 3.1 A New Definition of Representativeness

**Figure 2:**
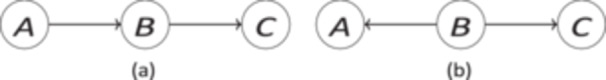
Causal structures of the sample (a) and the target population (b).

In this section, we present a new causally informed definition of representativeness based on the findings from Section 3, [Link]. The purpose of this definition is to specify as weak as possible conditions that are sufficient for generalising a finding in a sample to the target population. So far, we always implicitly assumed that the causal structure underlying the sample is the same as the one underlying the target population. This is, however, not at all a trivial assumption (for evidence see, e.g., [16]). To see why, let us discuss another simple example. Assume we are interested in *A*'s impact on *C*. Assume further that *B* mediates between *A* and *C*. Suppose we have a sample *s* in which all values of causes of *A, B, C* are distributed as in the target population. Finally, let us assume that the causal structure underlying *s* is the one shown in Figure 2a while the one underlying the target population is the one in Figure 2b.

Both structures are compatible with the exact same probability distributions. By assumption, the sample is representative in the sense of **RR**. However, results obtained from the sample *s* might get things horribly wrong about the target population. For example, assume we did a randomised controlled trial (RCT) based on our sample and found that *A* has a strong impact on *C*.^2^ Naturally, we infer that the same holds for the target population. However, since *A* is not causally relevant for *C* in the target population, *A* will give us no control at all over *C*. To avoid situations like these, representativeness requires that the causal structure underlying the sample is the same as the one underlying the target population. If we also take into account our findings from Section 3, [Link] about which variables need to be distributed equally, we arrive at the following definition:

**CR** A sample is representative of a target population w.r.t. *C*'s causal effect on *E* if (i) both share the same causal structure (and parameters^3^) and (ii) the values of all variables in **Z** are distributed in the sample as they are in the target population, where **Z** is the set of all variables *Z* different from *C* and *E* that are (ii.a) direct causes of *E* not lying on a directed path from *C* to *E* or (ii.b) direct causes of a variable *X* lying on a directed path from *C* to *E* not themselves lying on a directed path from *C* to *E*.

Our proposal pinpoints how the sample has to resemble the target population to guarantee that the obtained findings are generalisable to the latter. Consequently, **CR** can be seen as a natural development of both **GR** and **SR2**. Moreover, the definition is more precise and formal than its predecessors. Condition (i) reflects that the sample needs to have the same causal structure as the target population, while (ii) summarises our findings from Section 3, [Link]: Only variables need to be distributed in the sample as in the target population that could distort *C*'s impact on *E*. These are exactly the direct causes of *E* that are not effects of *C* (condition (ii.a)) as well as direct causes of variables on a path from *C* to *E* (condition (ii.b)).

**Example 1: Negative side‐effects of efavirenz in the population of Zimbabwe** ^4^

Efavirenz is a relatively cheap drug that prevents the replication of HIV and, therefore is effective in treating and preventing HIV/AIDS. The drug was tested in the U.S. and approved by the U.S. Food and Drug Administration in 1998. The degree to which its safety can be generalised beyond the population of the U.S. is crucial for the prospects of using the drug overseas (see [17]).

**Tested Effect:** Negative side‐effects of efavirenz

**Table:** 

**Target population**	**Sample population**
Population of Zimbabwe	Population of U.S.

**Assessment of causal similarity:**

The causal structure underlying the effects (including side effects) of Efavirenz is consistent across all humans. However, the prevalence of causally relevant factors varies among populations. For instance, a mutation in the CYP2B6 gene, which impedes the metabolization of Efavirenz, is much more common in Zimbabwe than in the U.S. (see [18]).

**Assessment of representativeness:**

Despite the shared underlying causal structure, differences in critical factors compromise the representativeness of results obtained in the U.S. for the population of Zimbabwe. However, it has been argued that dosage adjustment may effectively counter these differences [19], potentially making the drug as safe for use in Zimbabwe as it is in the U.S.

**Example 2: Effects of alcohol use disorder in humans**

Alcohol use disorder remains a prevalent and serious problem. Some of its aspects, such as underlying molecular mechanisms, remain poorly understood. Studies of the disorder in humans are changing due to practical and ethical reasons.

**Tested Effect:** Effects of alcohol use disorder

**Table:** 

**Target population**	**Sample population**
Humans	*Caenorhabditis elegans* worms

**Assessment of causal similarity:**

Studies have demonstrated that the causal structures underlying the physiological (e.g., [20]) and behavioural (e.g., [21]) effects of alcohol consumption, as well as Alcohol Use Disorder, are consistent in both humans and *Caenorhabditis elegans* worms. For example, corticotropin‐releasing factor receptors contribute to the development of alcohol‐seeking behaviour in both humans and *Caenorhabditis elegans* (see [21]). Some causally relevant factors, particularly those related to the size of the organisation, will need adjustment through tuning. However, it is likely that making these adjustments will not pose significant challenges.

**Assessment of representativeness:**

Given the current state of scientific knowledge regarding the causal structure underlying the effects of alcohol, it appears that results obtained in *Caenorhabditis elegans* will be representative of humans. For instance, research involving these worms will likely be valuable in studying the health consequences of Alcohol Use Disorder and testing pharmacological interventions aimed at treating it.

**Example 3: Efficiency of tumour necrosis factor blockers on sepsis**

Tumour necrosis factor (TNF) is believed to play a significant role in fatalities associated with sepsis. Consequently, medications that lower TNF are expected to improve the survival rates of patients suffering from sepsis. However, experimental findings have yielded surprisingly mixed results, revealing heterogeneity among sepsis cases (see e.g., [22], or [23]).

**Tested Effect:** Efficiency of TNF neutralisation.

**Table:** 

**Target population**	**Sample population**
Patients suffering from sepsis caused by *Escherichia coli* infection	Patients suffering from sepsis caused by *Streptococcus pneumoniae* infection

**Assessment of causal similarity:**

Despite the substantial similarity in symptoms, different cases of sepsis are caused by different types of bacteria (e.g., *Escherichia coli* or *Streptococcus pneumoniae*). Due to differences in bacterial characteristics, such as Gram type, these pathogens respond differently to TNF‐neutralising medications. This, in turn, results in distinct causal structures for each type of sepsis.

**Assessment of representativeness:**

Due to significant differences in the causal structure of different types of sepsis, it cannot be assumed that patients suffering from one type will be representative in how they respond to TNF neutralisation compared to patients with another type. This explains the observed mixed empirical results.

**Example 4: Penicillin toxicity in bats**

Scientists are striving to save the last population of critically endangered tropical species of bats dying due to a bacterial disease. They want to test if penicillin is a safe drug for bats.

**Tested Effect:** Penicillin Toxicity

**Table:** 

**Target population**	**Sample population**
Tropical Bats	Guinea Pigs

**Assessment of causal similarity:**

The gut flora of guinea pigs is primarily composed of gram‐positive organisms, which is atypical among mammals. Since the composition of intestinal flora causally influences the effects of penicillin consumption, it is reasonable to assume that the causal structure underlying the effects of penicillin in guinea pigs differs from that in bats (see [17, 24]).

**Assessment of representativeness:**

The causal structure underlying the effects of penicillin is likely different in guinea pigs than in bats. Consequently, studies using guinea pigs will not yield results that are representaive of bats.

## 4 Discussion

**CR** naturally translates into methodological instructions. To show that a sample is likely to be representative a scientist should: (a) present evidence suggesting that the causal structure is the same in the sample and target population, and (b) present evidence showing that causes that might distort the impact the variable intervened on has on the purported effect are distributed similarly in the sample and the target population. Evidence concerning (a) can come in the form of scientific theories describing how the tested cause will affect the members of the sample and target population. For example, if the best available genetic and physiological theories (see e.g., 2 and 3) predict that the causal structure is the same in the case of both populations, we have a strong reason to believe that (a) is satisfied in a given case. Justification for (b) should show that all (known) factors possibly distorting the intervention variable's purported effect are similarly distributed in both populations. This evidence can take the form of empirical results showing that the crucial properties are similarly distributed.

**Table:** 

Checklist for justification of representativeness		
No.	Item	
1.a	Present evidence demonstrating that the sample and the target population share the same underlying causal structure with respect to the tested variable *C*'s effect on *E*.	□
1.b	Present evidence demonstrating that the causal structures of the sample and target populations exhibit the same parameters representing the strength of the connections between related variables.	□
	For example, general biological or physiological theories have demonstrated that the underlying processes (e.g., metabolism of the tested substance) are the same in both populations. Evidence showing the presence of functionally analogous relevant organs in members of both populations further supports this similarity and so on.	
2.a	Present evidence demonstrating that the values of direct causes of *E* that do not lie on a directed path from *C* to *E*—that is, causes of the tested effect *E* that are not caused by *C*—are the same in the sample as in the target population.	□
2.b	Present evidence demonstrating that the values of direct causes of a variable *X* that are not lying on a directed path from *C* to *E* are the same in the sample as in the target population.	□
	For example, empirical evidence showing that the values of causes of the tested effect (other than the tested cause) and the external causes of other variables on the path from *C* to *E* are comparable in both populations.	
3.	Counterbalance any remaining significant differences in the parameters and variables mentioned above.	□
	For example, differences in body mass between the members of both populations can be accounted for by adjusting the dosage of the tested substance.

Another implication of **CR** is that there are two distinct ways in which a sample may fail to be representative. Firstly, the causal structure that connects (or disconnects) studied variables may be different in the sample and target population. Secondly, the sample may be nonrepresentative because some of the possible distorting variables are not distributed equally in both populations. It seems that the failure of the second kind may be amended, for example, by taking into account unbalances or counteracting them. As sketched in Example 2, reducing the dose of Efavirenz administered to the population in Zimbabwe may lower the prevalence of negative side effects to levels observed in the U.S. while preserving most of its antiviral efficacy (see [19] and [17] for discussion). At the same time, the failures of replicability of the first type seem to be harder to amend and perhaps require composing a new sample.

What are the limitations of **CR**? Having a sound and precise definition of representability is one thing, how to ensure representability in practice is another. It is a very difficult task to achieve certain knowledge about causal structure or to exclude confounding factors with certainty. However, **CR** provides more guidance for how to maximise certainty or track uncertainty. There is a vast literature on inferring causal structure based on observational data and there are ways to account for confounders, selection bias, and other disturbing factors (e.g., [25, 26, 27]). Though causal search algorithms will usually not output a single causal structure, but rather a set of structures compatible with the data, this might still be useful. Such results can be complemented by expert evaluations or background knowledge such as information about temporal order. Thus, even though the correct causal structure as well as disturbing factors can usually not be identified with certainty, **CR** might still guide scientists in evaluating the suitability of a sample for an inference task or choosing the most promising sample when possible. Among other things, **CR** provides awareness for the causal pitfalls that come with simply ignoring its implications.

What are the consequences of the new conceptualisation for the relativity of representativeness? In line with Rudolph et al. [1], **CR** predicts that a sample is representative only of a specific target population; therefore, this target population must be well described. Moreover, it implies that representativeness is relative to the variable intervened on and the tested effect(s). This is plausible: A sample may be representative of a target population w.r.t. one effect but not w.r.t. another. For example, the *Caenorhabditis elegans* worms appear to be representative of humans in terms of the effects of alcohol consumption but not with respect to the effects of adaptation to low temperatures. In contrast to [5] and [28], **CR** does not imply that representativeness is relative to time and location. A sample will remain representative as long as the relevant causal structure and causally relevant properties in the target population remain unchanged. Finally, we would like to discuss the broader implications of the results of this paper. We demonstrated that knowledge of the causal structure can be used to guide and simplify the search for a representative sample. We do not need to replicate all features of the target population in the sample (as in **RR**), but only those that could causally distort the intervened on variable's impact on the purported effect. At the same time, we do not claim that the causal approach is the only way to go. Another plausible approach would be to redefine the notion of similarity (between the sample and the target population) in a mathematically precise noncausal manner, for example, along the lines of Douven et al. [29]. Regardless of the specific method chosen, we are confident that we have demonstrated the viability of a more formal approach.

## Conflicts of Interest

The authors declare no conflicts of interest.

## Data Availability

The authors have nothing to report.

## Endnotes

1 This is an advantage over more traditional approaches such as Rubin's causal model. We chose CBNs rather than SEMs because they are more general in that they can also work with nonparametric assumptions, that is, without knowledge of functions and distributions.

2 For details on the difference between observation and intervention (as in an RCT) see, for example, (Pearl 2000).

3 A variable's parameters are the probabilities of its values given all possible value combinations of its direct causes.

4 The source of this example is (Park et al. 2023).

## References

[1] J. E. Rudolph , Y. Zhong , P. Duggal , S. H. Mehta , and B. Lau , “Defining Representativeness of Study Samples in Medical and Population Health Research,” BMJ Medicine 2 (2023): e000399. 37215072 10.1136/bmjmed-2022-000399 PMC10193086

[2] D. R. Cox , Principles of Statistical Inference (Cambridge University Press, 2006).

[3] J. M. Bernardo , “The Concept of Exchangeability and Its Applications,” Far East Journal of Mathematical Sciences 4 (1996): 111–121.

[4] K. J. Rothman , J. E. Gallacher , and E. E. Hatch , “Why Representativeness Should Be Avoided,” International Journal of Epidemiology 42, no. 4 (2013): 1012–1014. 24062287 10.1093/ije/dys223 PMC3888189

[5] E. A. Nohr and J. Olsen , “Commentary: Epidemiologists Have Debated Representativeness for More Than 40 Years Has the Time Come to Move On?,” International Journal of Epidemiology 42, no. 4 (2013): 1016–1017. 24062289 10.1093/ije/dyt102

[6] J. M. Elwood , “Commentary: On Representativeness,” International Journal of Epidemiology 42, no. 4 (2013): 1014–1015. 24062288 10.1093/ije/dyt101

[7] L. Richiardi , C. Pizzi , and N. Pearce , “Commentary: Representativeness Is Usually Not Necessary and Often Should Be Avoided,” International Journal of Epidemiology 42, no. 4 (2013): 1018–1022. 24062290 10.1093/ije/dyt103

[8] J. Last , A Dictionary of Epidemiology (Oxford University Press, 1983).

[9] M. Porta , A Dictionary of Epidemiology (Oxford University Press, 2016).

[10] J. Pearl , Causality, 1st ed. (Cambridge University Press, 2000).

[11] P. Spirtes , C. Glymour , and R. Scheines , Causation, Prediction, and Search, 1st ed. (Springer, 1993).

[12] J. Pearl and E. Bareinboim , “External Validity and Transportability: A Formal Approach,” 2011 JSM Proceedings (2011): 157–171.

[13] P. W. G. Tennant , E. J. Murray , K. F. Arnold , et al., “Use of Directed Acyclic Graphs (DAGS) to Identify Confounders in Applied Health Research: Review and Recommendations,” International Journal of Epidemiology 50, no. 2 (2021): 620–632. 33330936 10.1093/ije/dyaa213 PMC8128477

[14] M. Webster‐Clark and A. Breskin , “Directed Acyclic Graphs, Effect Measure Modification, and Generalizability,” American Journal of Epidemiology 190, no. 2 (2021): 322–327. 32840557 10.1093/aje/kwaa185

[15] M. Webster‐Clark , R. K. Ross , A. P. Keil , and R. W. Platt , “Variable Selection When Estimating Effects in External Target Populations,” American Journal of Epidemiology 193, no, 8 (2024): 1176–1181. 38629587 10.1093/aje/kwae048 PMC11299018

[16] N. Cartwright and J. Hardie , Evidence‐Based Policy: A Practical Guide to Doing It Better (Oxford University Press, 2012).

[17] A. Park , D. Steel , and E. Maine , “Evidence‐Based Medicine and Mechanistic Evidence: The Case of the Failed Rollout of Efavirenz in Zimbabwe,” Journal of Medicine and Philosophy: A Forum for Bioethics and Philosophy of Medicine 48 (2023): 348–358. 10.1093/jmp/jhad019 PMC10281362 37137159

[18] C. Masimirembwa , C. Dandara , and P. D. C. Leutscher , “Rolling out Efavirenz for HIV Precision Medicine in Africa: Are We Ready for Pharmacovigilance and Tackling Neuropsychiatric Adverse Effects?,” Omics: A Journal of Integrative Biology 20, no. 10 (2016): 575–580. 27627692 10.1089/omi.2016.0120

[19] C. Nyakutira , D. Röshammar , E. Chigutsa , et al., “High Prevalence of the cyp2b6 516g→t(*6) Variant and Effect on the Population Pharmacokinetics of Efavirenz in HIV/AIDS Outpatients in Zimbabwe,” European Journal of Clinical Pharmacology 64 (2008): 357–365. 18057928 10.1007/s00228-007-0412-3

[20] X. Yu , W. Zhao , J. Ma , X. Fu , and Z. J. Zhao , “Beneficial and Harmful Effects of Alcohol Exposure on *Caenorhabditis elegans* Worms,” Biochemical and Biophysical Research Communications 412, no. 4 (2011): 757–762. 21871869 10.1016/j.bbrc.2011.08.053

[21] C. Salim , A. K. Kan , E. Batsaikhan , E. C. Patterson , and C. Jee , “Neuropeptidergic Regulation of Compulsive Ethanol Seeking in C. elegans,” Scientific Reports 12 (2022): 1804. 35110557 10.1038/s41598-022-05256-1 PMC8810865

[22] J. A. Lorente and J. C. Marshall , “Neutralization of Tumor Necrosis Factor in Preclinical Models of Sepsis,” Shock 24 (2005): 107–119. 16374382 10.1097/01.shk.0000191343.21228.78

[23] J. C. Marshall , “Why Have Clinical Trials in Sepsis Failed?,” Trends in Molecular Medicine 20, no. 4 (2014): 195–203. 24581450 10.1016/j.molmed.2014.01.007

[24] R. H. Green , “The Association of Viral Activation With Penicillin Toxicity in Guinea Pigs and Hamsters,” Yale Journal of Biology and Medicine 47 (1974): 166–181. 4446629 PMC2595098

[25] T. Richardson and P. Spirtes , “Ancestral Graph Markov Models,” Annals of Statistics 30, no. 4 (2002): 962–1030.

[26] P. Spirtes , C. Meek , and T. Richardson , “An Algorithm for Causal Inference in the Presence of Latent Variables and Selection Bias,” Proceedings of the 11th Conference on Uncertainty in Artificial Intelligence (Morgan Kaufman, 1999), 499–506.

[27] J. Zhang , “Reasoning With Ancestral Graphs,” Journal of Machine Learning Research 9 (2008): 1437–1474.

[28] J. Olsen , “Random Sampling—Is It Worth It?,” Paediatric and Perinatal Epidemiology 27, no. 1 (2013): 27–28. 23215707 10.1111/ppe.12020

[29] I. Douven , S. Elqayam , P. Gärdenfors , and P. Mirabile , “Conceptual Spaces and the Strength of Similarity‐Based Arguments,” Cognition 218 (2022): 104951. 34801861 10.1016/j.cognition.2021.104951

